# A Study on the Relevance of Glucose-6-Phosphate Dehydrogenase Level Screening in Patients with Rheumatic Diseases Prior to Initiating Treatment With Hydroxychloroquine

**DOI:** 10.7759/cureus.9519

**Published:** 2020-08-02

**Authors:** Irina Abramova, Kyle Park, Carol Hosny, Swosty Tuladhar, QingPing Yao, Asha Patnaik

**Affiliations:** 1 Division of Rheumatology, Department of Internal Medicine, Stony Brook University, Stony Brook, USA; 2 Department of Internal Medicine, Stony Brook University, Stony Brook, USA

**Keywords:** hydroxychloroquine, sulfasalazine, glucose-6-phosphate-dehydrogenase deficiency (g6pd), systemic lupus erythematosus (sle), rheumatoid arthritis (ra)

## Abstract

Objective

Drug-induced hemolytic anemia can occur in patients with glucose-6-phosphate-dehydrogenase (G6PD) deficiency. The practice of G6PD-deficiency screening in the rheumatology field has been inconsistent. This study aimed to determine the utility of screening prior to the initiation of hydroxychloroquine and/or sulfasalazine in rheumatology patients in the ambulatory clinics at Stony Brook University Hospital, New York.

Methods

We conducted a retrospective chart review of cases of rheumatic diseases that were screened for G6PD deficiency at Stony Brook University Hospital ambulatory clinics. Demographic details and relevant clinical and laboratory data of the patients were collected. The data from similar studies in the literature were searched for and reviewed.

Results

This study consisted of 228 patients with systemic lupus erythematosus (SLE), rheumatoid arthritis (RA), and Sjögren’s syndrome. Among those patients, 94.7% received hydroxychloroquine, sulfasalazine, or dapsone; 41% (89/228) of patients were screened for G6PD deficiency, and the majority of them were on treatment with hydroxychloroquine. Of those patients, 7.9% (five Caucasians and two African Americans) were found to have G6PD deficiency, and two of the G6PD-deficient patients received hydroxychloroquine. There was no incidence of hemolytic anemia documented in any of the seven patients with G6PD deficiency. We reviewed the literature and found three similar studies of patients receiving hydroxychloroquine with no reported hemolytic anemia from different medical centers in the US, and the frequency of G6PD deficiency reported in these studies was 1.4%, 4.0%, and 4.2%, respectively.

Conclusions

Our study suggests that the frequency of G6PD deficiency in our rheumatic population is similar to that of the general population, and the risk of hemolytic anemia in G6PD deficiency associated with hydroxychloroquine is extremely rare. Hence, G6PD screening may not be recommended prior to starting treatment with hydroxychloroquine.

## Introduction

**Figure 1:**
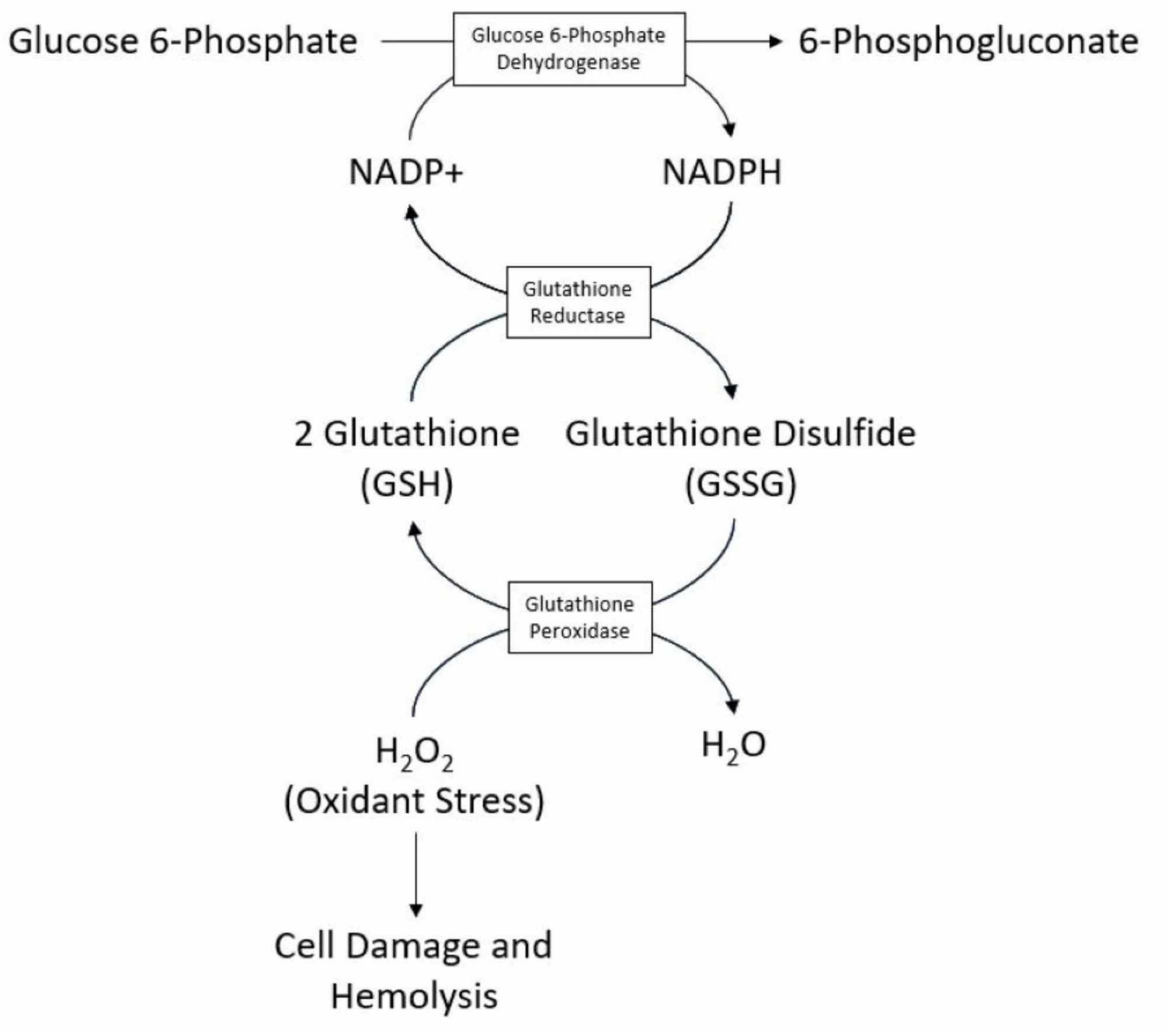
Mechanism of G6PD in preventing hemolysis in the setting of oxidant stress* *[8] G6PD: glucose-6-phosphate-dehydrogenase; NADP+: nicotinamide adenine dinucleotide phosphate; NADPH: nicotinamide adenine dinucleotide phosphate hydrogen

Glucose-6-phosphate-dehydrogenase (G6PD) deficiency is the most common enzymatic disorder of the red blood cells. It is an X-linked hereditary disease and thus mainly affects males. Its frequency varies among different ethnicities, with the highest incidence reported among Kurdish Jews, Saudis, and African Americans [1]. G6PD prevents hemolysis in the setting of oxidant stress by the production of nicotinamide adenine dinucleotide phosphate hydrogen (NADPH) (Figure 1). In patients with certain variants of G6PD deficiency, hemolytic anemia can develop due to oxidant stress during the neonatal period, infection, or such exogenous agents as fava beans and certain medications [2]. In the field of rheumatology, concerns have been raised about several medications for triggering hemolytic anemia in patients with G6PD deficiency.

The FDA's drug labels for hydroxychloroquine [3], sulfasalazine [4], and dapsone [5] list the risk of hemolytic anemia in patients with G6PD deficiency. However, there are no guidelines currently available for screening of patients for G6PD deficiency prior to starting the above medications. Clinically, there is an inconsistent practice among rheumatologists of testing G6PD levels in patients before initiating drugs like hydroxychloroquine.

Concerns regarding hemolytic anemia associated with hydroxychloroquine in patients with G6PD deficiency mainly resulted from studies of antimalarials, particularly 8-aminoquinolines such as primaquine, for the treatment of malaria. Studies have found that the risks of hemolytic anemia with 8-aminoquinolines are dose-dependent and G6PD subtype-dependent [6,7]. However, only a few studies have been done on the incidence of hemolytic anemia in G6PD-deficient patients taking hydroxychloroquine for rheumatic diseases.

Our study aimed to report the frequency of G6PD deficiency in the rheumatic disease population. It was based on G6PD testing prior to the initiation of medications including hydroxychloroquine in our hospital coupled with a review of the relevant literature.

## Materials and methods

We conducted a retrospective electronic medical record (EMR) review using our EMR supported by Cerner System (Cerner Corporation, Kansas City, MO). Our subjects were patients attending the ambulatory rheumatology clinics of Stony Brook University Medical Center between July 2013 and December 2016. The following selection criteria were used: patients who were 18 years and older with systemic lupus erythematosus (SLE), rheumatoid arthritis (RA), or Sjögren’s syndrome. The International Classification of Diseases, Ninth Revision (ICD9) codes were used to capture the diagnosis in the EMR. These patients were treated with either hydroxychloroquine, sulfasalazine, or dapsone. G6PD testing results of the patients were recorded if those were available on their electronic charts. Patients were determined to have G6PD deficiency if the G6PD level was less than 7.0 U/g as reported in commercial laboratories. For patients meeting these criteria, their demographic data, rheumatic diseases, age at diagnosis, and rheumatic medications were recorded (Table 1). For statistical analysis, descriptive statistics and chi-square/Fisher's exact tests were used. This study was approved by the Institutional Research Board of Stony Brook University, New York. The procedures followed in this study were in accordance with the ethical standards of the responsible committee on human experimentation and with the Helsinki Declaration of 1975, as revised in 2000 and 2008.

Literature was searched using the MEDLINE® and PubMed databases with such indexing terms as "Systemic Lupus Erythematosus, Rheumatoid Arthritis, Sjögren’s Syndrome, hydroxychloroquine, and G6PD". Relevant publications between 1970 and November 2018 about G6PD deficiency and these rheumatic diseases were reviewed for data synthesis and analysis. Among the literature reviewed, three studies met the search criteria, which are discussed in Table 2.

## Results

Based on the EMR chart review of the 228 patients with rheumatic diseases, 216 (94.7%) patients were found to be on hydroxychloroquine, sulfasalazine, and/or dapsone in this study. Of those, 89 (41%) patients were screened for G6PD deficiency prior to starting treatment by more than one provider. Seven (7.9%) of those patients were found to be G6PD-deficient. The mean age of the G6PD-deficient patients was 34.9 years, and they included five (71%) Caucasians of Italian descent and two (29%) African Americans. An overwhelming majority of patients (213 of 228) with G6PD testing had SLE (93.9%); RA was found in 30 patients (13.4%), and/or Sjögren’s syndrome in 28 patients (12.2%). Almost all patients (97.6%) tested for G6PD were treated with oral hydroxychloroquine 200 mg twice daily. The mean disease duration in patients with G6PD deficiency was 11.1 years.

Of the seven patients with G6PD deficiency, two were started on hydroxychloroquine. There was no incidence of hemolytic anemia documented in all seven patients with G6PD deficiency. The demographics and clinical data of patients with G6PD testing are shown in Table 1.

**Table 1:** Demographics and clinical data of patients tested for G6PD and those with deficiency (n=89) G6PD: glucose-6-phosphate-dehydrogenase; IQR: interquartile range; IBD: inflammatory bowel disease

Variables	G6PD deficiency		
Gender	No (n=82)	Yes (n=7)	P-value
Male	2 (2.44%)	2 (28.57%)	0.0298
Female	80 (97.56%)	5 (71.43%)	
Age			
Median (IQR)	43.3 (22.3)	34.9 (33.4)	0.9332
Race			
White/Caucasian	45 (55.56%)	5 (71.43%)	0.7824
Black/African American	16 (19.75%)	2 (28.57%)	
Hispanic/Latino	8 (9.88%)	0 (0.0%)	
Other	12 (14.81%)	0 (0.0%)	
Age at diagnosis (65 missing)			
Median (IQR)	35 (24)	25 (15)	0.0890
Duration of disease in years (65 missing)			
Median (IQR)	4.6 (4.6)	11.1 (21.3)	0.2984
Systemic lupus erythematosus			
No	5 (6.10%)	1 (14.29%)	0.3974
Yes	77 (93.90%)	6 (85.71%)	
Psoriatic arthritis			
No	81 (98.78%)	7 (100.0%)	1.0000
Yes	1 (1.22%)	0 (0.0%)	
Ankylosing spondylitis			
No	81 (98.78%)	7 (100.0%)	1.0000
Yes	1 (1.22%)	0 (0.0%)	
IBD arthropathy			
No	82 (100.0%)	7 (100.0%)	Not applicable
Yes	0 (0.0%)	0 (0.0%)	
Rheumatoid arthritis			
No	71 (86.59%)	7 (100.0%)	0.5899
Yes	11 (13.41%)	0 (0.0%)	
Sjögren’s syndrome			
No	72 (87.80%)	6 (85.71%)	1.0000
Yes	10 (12.20%)	1 (14.29%)	
Hydroxychloroquine use (1 missing)			
No	2 (2.44%)	5 (71.43%)	<0.0001
Yes	80 (97.56%)	2 (28.57%)	
Sulfasalazine			
No	81 (98.78%)	7 (100.0%)	1.0000
Yes	1 (1.22%)	0 (0.0%)	
Dapsone			
No	82 (100.0%)	7 (100.0%)	Not applicable
Yes	0 (0.0%)	0 (0.0%)	
Duration of drug use in years (94 missing)			
Median (IQR)	3.4 (2.6)	4.4 (6.0)	0.9502

## Discussion

The literature data and comparison with our data

In our literature review, we found only three studies that investigated the risk of hemolytic anemia in G6PD-deficient patients on hydroxychloroquine.

One study from the Duke University Medical Center in 2017 found that out of 275 patients taking hydroxychloroquine with measured G6PD levels, only 11 (4.0%) patients were determined to have G6PD deficiency. Among those, 11 patients were African Americans with nine females and two males. Only two patients had episodes of hemolysis, which did not occur during hydroxychloroquine therapy [9]. In another study from Maryland General Hospital in 2011, of the 170 patients with the diagnosis of RA and/or SLE, 24 patients had serum G6PD levels tested and only one African American was found to be G6PD-deficient with no adverse clinical events despite long-term use of hydroxychloroquine (Presentation: Mercedes Q, Dowell S, Sharma A, Flores R, Hochberg M, Mikdashi J, Rus V. Presentation 2077: The Use of Hydroxychloroquine in Patients with Rheumatoid Arthritis and Systemic Lupus Erythematosus: To Check or Not to Check Glucose-6-Phosphate Dehydrogenase Levels Prior to Its Initiation in Everyday Rheumatology Practice. American College of Rheumatology and Association of Rheumatology Health Professionals Scientific Meeting; 2011). In another study from Jacobi Medical Center, 36 out of 2,605 (1.4%) patients with rheumatic diseases were found to be deficient in G6PD, and 18 of those patients received hydroxychloroquine without the development of hemolytic anemia (Presentation: Saldarriaga MM, Ramirez de Oleo IE, Johnson B. Presentation 2139: Retrospective Study: Association of Hydroxychloroquine Use and Hemolytic Anemia in Patients with Low Levels of Glucose-6-Phosphate Dehydrogenase (G6PD). American College of Rheumatology and Association of Rheumatology Health Professionals Scientific Meeting; 2011). A comparison of results from these studies and those from the current study is detailed in Table 2.

**Table 2:** Comparison of studies investigating the risk of hemolytic anemia in patients with G6PD deficiency and rheumatic diseases associated with the use of hydroxychloroquine *Subjects with rheumatic disease in the study tested for G6PD level; **Hemolytic anemia did not occur during HCQ therapy; ***In the study by Saldarriga et al., only the demographics of G6PD-deficient individuals were described G6PD: glucose-6-phosphate-dehydrogenase; SLE: systemic lupus erythematosus; RA: rheumatoid arthritis; HCQ: hydroxychloroquine

Variables	Abramova et al. (current study)	Mohammad et al. [9]	Mercedes et al. (presentation: Mercedes, 2011)	Saldarriaga et al. (presentation: Saldarriaga, 2011)
Institution	Stony Brook University Hospital	Duke University Medical Center	Maryland General Hospital	Jacobi Medical Center
Sample size*	89	275	24	2,605
G6PD deficiency, n (%)	7 (7.9%)	11 (4.0%)	1 (4.2%)	36 (1.4%)***
Gender, n (%)				
Female	5 (7.1%)	9 (82%)	Not described	11 (61%)***
Male	2 (2.9%)	2 (18%)	Not described	7 (39%)***
Race. n (%)				
African American	2 (2.9%)	11 (100%)	1 (100%)	10 (56%)***
Caucasian	5 (7.1%)	0 (0%)	0 (0%)	Not described
Others	0 (0%)	0 (0%)	0 (0%)	Not described
Autoimmune disease, n (%)				
SLE	6 (86%)	7 (64%)	Not described	7 (39%)***
RA	0 (0%)	2 (18%)	Not described	5 (28%)***
Sjögren’s syndrome	1 (14%)	0 (0%)	Not described	Not described
Others	0 (0%)	1 (9.1%)	Not described	Not described
Hydroxychloroquine use, n (%)	2 (2.9%)	11 (100%)	Not described	18 (50%)
Patients with hemolytic anemia/patients on HCQ therapy with G6PD deficiency, n (%)	0/2 (0%)	2/11** (18%)	0/1 (0%)	0/18 (0%)

Discussion of results from this study

In our single-center retrospective study, we found that the frequency of G6PD deficiency was 7.9% in the patient population with rheumatic diseases at Stony Brook University Hospital, Long Island, New York. Based on the reported study results from three different medical centers, the frequency of G6PD deficiency was 4.0% [9], 4.2% (presentation: Mercedes, 2011), and 1.4% (presentation: Saldarriaga, 2011) respectively. Together, these results are largely within the range of 0.2-7.2% reported in the general American population [10].

G6PD deficiency is an X-linked hereditary disease and mainly affects males, but females can also be affected under certain circumstances. In our study, we identified more female patients with G6PD deficiency than males. This is likely due to the lupus population being predominantly female. In general, G6PD deficiency is more commonly associated with the African American population [1], and this finding is also supported by some previous studies on G6PD deficiency in the rheumatic disease population [9] (presentation: Mercedes, 2011; presentation: Saldarriaga, 2011). However, in our study, five out of seven patients with G6PD deficiency were identified to be Caucasian.

Numerous medications have been reported to cause anemia in G6PD-deficient individuals. For example, in a review by Youngster et al. in 2010, robust evidence supporting the association of drug-induced hemolysis with medications such as dapsone, methylene blue, nitrofurantoin, phenazopyridine, primaquine, rasburicase, and toluidine blue was reported [11].

Primaquine has been strongly associated with hemolytic anemia in patients with G6PD deficiency. The risk of hemolytic anemia with primaquine is dose-dependent and G6PD subtype-dependent [6,7]. According to a 2014 WHO publication on the safety of 8-aminoquinoline antimalarial medications, there has been a total of 190 reports of severe adverse reactions associated with primaquine, all of which occurred in patients with confirmed or likely G6PD deficiency [12]. Reports of severe hemolysis attributed to primaquine use have been relatively rare, with only 12 reported deaths due to hemolysis associated with primaquine over the last six decades [13]. The mechanism by which primaquine, an 8-aminoquinoline, causes hemolytic anemia in G6PD-deficient individuals is hypothesized to be due to the generation of hydrogen peroxide in erythrocytes from the drug [13], thereby increasing oxidant stress and predisposing the individual to hemolysis as described in Figure 1. However, hydrogen peroxide was not detected in erythrocytes after the addition of 4-aminoquinolines [13] that are hydroxychloroquine and chloroquine, and this may explain the safety of these drugs relative to primaquine in G6PD-deficient individuals.

Dapsone is another medication that has been shown to have a clear association with hemolytic anemia in G6PD-deficient individuals. Dapsone has been known to cause mild hemolytic anemia in normal individuals [14] and hemolytic anemia at a more significant rate and intensity in G6PD-deficient individuals [15] since the 1960s. Several, more recent clinical trials of dapsone-containing drug regimens for the treatment of malaria have confirmed a significant rate of hemolytic anemia in G6PD-deficient individuals receiving dapsone-containing treatments, with many patients having significant hemolytic reactions requiring blood transfusions [16-18]. The mechanism of dapsone-induced hemolytic anemia has been proposed to be due to N-hydroxy metabolites of dapsone, which are direct hemolytic agents [19].

On the contrary, the evidence for the association between sulfasalazine and hemolytic anemia in G6PD-deficient individuals is scarce. There have been individual case studies dating back to the 1960s and 1970s on hemolytic anemia possibly associated with the administration of sulfasalazine in individuals with and without G6PD deficiency [20,21]. However, there have not been any large studies on the incidence of hemolytic anemia in G6PD-deficient individuals receiving sulfasalazine.

Overall, our study and the data in the literature suggest that G6PD testing prior to and/or during the initiation of patients on hydroxychloroquine may not be recommended given the low incidence of G6PD deficiency in that patient population and the very low risk of anemia in the patients treated with the drug. Instead, we would recommend that routine monitoring of complete blood counts may be sufficient.

Our study has several limitations. This was a retrospective study with relatively small sample size, and it was done only at our institution; thus it resulted in limited power. Another limitation of our study is that its results cannot be extrapolated to other SLE populations that may differ in their geographical ancestry and risk for G6PD deficiency. Due to the lack of data in our study, sulfasalazine and dapsone and G6PD/anemia were not adequately addressed.

The results of this study were presented at the American College of Rheumatology's 2018 ACR/ARHP Annual Meeting (meeting abstract: Abramova I, Tuladhar S, Park K, et al. Revisit an Old Question: Should Glucose-6-Phosphate Dehydrogenase Level be Checked in Patients with Rheumatic Diseases Prior to Initiating Certain Drugs? American College of Rheumatology and Association of Rheumatology Health Professionals Annual Meeting; 2018).

## Conclusions

Based on our findings, the frequency of G6PD deficiency in the rheumatic disease population is similar to that of the general population. The risk of hemolytic anemia in G6PD deficiency associated with hydroxychloroquine is rare. G6PD testing prior to the initiation of patients on hydroxychloroquine is not indicated due to the low incidence of G6PD deficiency in that patient population and a very low risk of anemia in the patients treated with the drug. Based on this study, we do not recommend G6PD screening prior to starting treatment with hydroxychloroquine.
